# Towards universal health coverage and sustainable financing in Afghanistan: progress and challenges

**DOI:** 10.7189/jogh.08.02038

**Published:** 2018-12

**Authors:** Ariel Higgins-Steele, Farhad Farewar, Fazil Ahmad, Abdul Qadir, Karen Edmond

**Affiliations:** 1 UNICEF, Kabul, Afghanistan; 2 Ministry of Public Health, Kabul, Afghanistan

Afghanistan has made impressive progress in introducing primary health care across the country over the last fifteen years. In the face of the diverse challenges ranging from persistent insecurity, conservative social norms, weak public financing, and reducing donor aid [1], Afghanistan is arguably among one of the most challenging contexts to achieve universal health coverage (UHC). Therefore, it is important to consider additional avenues towards UHC while building on the progress in coverage and financing of primary health care to date.

The right to health is considered a fundamental human right [2] and UHC has gained prominence globally as a goal for countries in the Sustainable Development Goals (SDGs). UHC is defined by the World Health Organization (WHO) as: “access to key promotive, preventive, curative and rehabilitative health interventions for all at an affordable cost, thereby achieving equity in access. A key element is cost, and the principle of financial-risk protection which ensures that the cost of care does not put people at risk of financial catastrophe. A related objective of health-financing policy is equity in financing: households contribute to the health system on the basis of ability to pay” [3]. This definition incorporates themes identified in UHC literature, specifically: access to care or insurance, coverage, packages of services, rights-based approaches, and social and economic risk protection [4]. Critical elements underpinning UHC include: an efficient, resilient health system; affordable care and a system of financing health care that does not impoverish users; access to essential medicines and technologies; health workers who are motivated, and sufficient in number and skills; efficient, functional administrative and governance arrangements; and transparency in tracking progress and achieving equity [5].

## CURRENT STATUS AND PROGRESS OF UNIVERSAL HEALTH COVERAGE IN AFGHANISTAN

In 2016, the Government of the Islamic Republic of Afghanistan (GoIRA) committed to achieving the SDGs, with leadership for coordination, implementation, and reporting assigned to its Ministry of Economy. Target 3.8 commits Afghanistan to: “achieving universal health coverage, access to quality essential health-care services and access to safe, effective, quality and affordable essential medicines and vaccines for all.” This target is to be measured by indicators including: coverage of tracer interventions (eg, child full immunization, antiretroviral therapy, tuberculosis treatment, hypertension treatment, skilled attendant at birth) and, the proportion of the population protected against catastrophic/ impoverishing out-of-pocket health expenditure.

Though Afghanistan has made progress in some of these areas in the last fifteen years, the country still has among some of the worst levels of coverage of basic health interventions in the world for baseline indicators for its new commitment to SDG 3.8. For example, less than half of children (45.7%) are fully immunized and the country is one of the last countries to not yet have eliminated wild poliovirus [6]. Obstetric care also remains poor. Only half of all women in Afghanistan deliver at a health facility (48.1%) and only half of all women have a skilled attendant (doctor or midwife assisting with the delivery) at the birth (50.5%) [6].

There are known challenges in using SDG indicators to measure UHC (eg, availability of reliable data, tracers are only indicative of some areas of UHC). These are major constraints within low income countries including Afghanistan. However, there is much to be gained in benchmarking against a set of standard indicators globally. Additionally, taken at their baseline rates, these indicators highlight crucial areas for focus in Afghanistan.

Afghanistan is still struggling to extend access to basic health services. For rural populations, one survey conducted in 2014 indicated that 90% of the population have access to a health post or a public clinic within two hours [7]. However, only three quarters of the population can reach a referral hospital or a private clinic within this time and access is often impeded by harsh conditions in the winter months and due to conflict and violence. Rural populations also tend to pay around nine times more than the urban population for a one-way trip to a health facility [7]. For vulnerable groups, perceived availability of health care and experience with coverage has not greatly improved over the last ten years [8]. Indeed there is much concern that Afghanistan will not reach SDG 3 focused on health and well-being.

Afghanistan’s level of public sector financing is currently 5% of total health expenditure (THE), and was estimated to be only 97 million USD out of the total 1992 million THE in 2014 [9]. This is significantly lower than the 10% reported for other low-income countries globally [10]. In addition, out-of-pocket expenditures (OOP) are substantially higher in Afghanistan than many other low income countries [10]. More than two-thirds (71.8%) of THE – which includes public and private spending – are paid out of pocket. This places a high burden on households and has remained unchanged in recent years [9]. This compares to 42% on average for other low income countries [10]. In contrast, international donors provide 20.8% of the THE (USD 312.5 million) [9].

**Figure:**
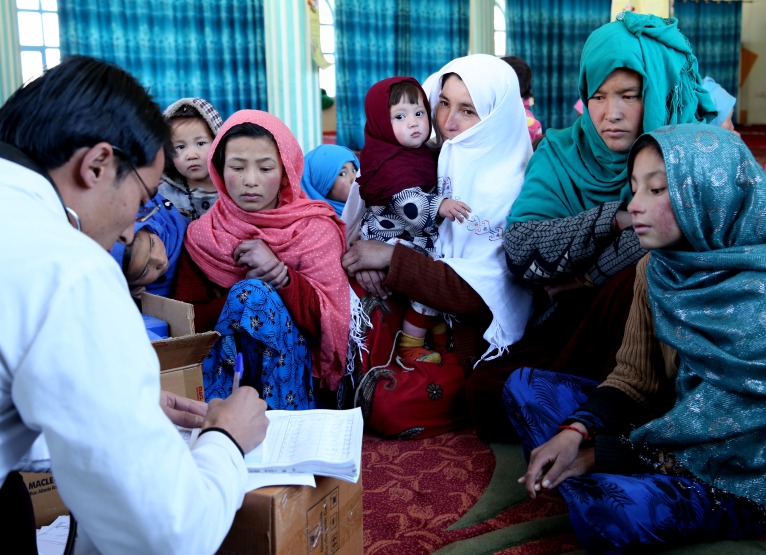
Photo caption: A nurse from a mobile health team provides health advice to women and girls in a remote village, Ali Beig, in Bamyan province Afghanistan (UNICEF Afghanistan, 2016, Karimi; used with permission)

## POSSIBLE APPROACHES TO ACHIEVING UNIVERSAL HEALTH COVERAGE IN AFGHANISTAN

Given Afghanistan has already implemented an evidence-based package of primary health care services, to expand coverage Afghanistan now needs both innovative access models and improved health care financing that can be melded into the current climate of increasing conflict across the country.

There is general agreement that primary health care must be the focus of UHC in Afghanistan [11]. However, there are differing views on the best approach to move towards universal coverage of primary health care services. A traditional approach is to promote a limited number of basic, life-saving interventions to the population, called the selective health care model [12], which Afghanistan has been implementing. Another approach used by low- and middle-income countries (LMICs), involved structural adjustments in the 1980s which included increasing private insurance schemes and user fees; a re-organisation of the health system with decentralization to lower levels of the health system; staff layoffs and streamlined health administration; altering the pharmaceutical sector towards more marketization; and privatization and out-contracting of health services [13]. However, structural adjustments have not been attempted in Afghanistan due to difficulties with centralized governance and monitoring.

Afghanistan underwent a substantial reform of health services just over fifteen years ago. Confronted by an uncoordinated and poorly performing health care system following the fall of the Taliban, the Ministry of Public Health instituted a number of key policies, the most important of which were: pooling donor funds through the Afghanistan Reconstruction Trust Fund (ARTF); defining a package of priority health services known as the basic package of health services (BPHS); purchasing or contracting with non-governmental organizations (NGOs) (called the ‘contracting out’ model) to deliver this package of primary health care services; and monitoring and evaluation of health sector performance [14,15]. The BPHS includes key primary health interventions for all population groups, with particular emphasis on care for women and children. Services are supposed to be free of cost for all people; however, direct and indirect costs associated with health care such as transport, informal payments at private facilities and use of public facilities contribute to persistent high out-of-pocket expenditures.

The first years of implementation of the BPHS resulted in substantial improvements compared to the very low 2003 baseline. There has also been substantial progress in other social service sectors including birth registration and child protection [15]. Reviews have led to new interventions added into Afghanistan’s BPHS package – eg, chlorhexidine for newborn cord care, zinc for treatment of childhood diarrhoea, etc. – as well as focus on areas where access remains a challenge due to insecurity or difficult geographic terrain.

Innovative access models include investment in mobile health teams, outreach from fixed centres, improving literacy and incentives or salaries for community health workers, strengthened governance of the ‘contracting out’ model of nongovernment organisations, and monitoring through the new citizen charter movement [16]. These efforts seek to expand preventive and curative functions of the health system based on empirical evidence, as well as expand geographic reach.

Despite increases in coverage in Afghanistan, high out-of-pocket expenditures indicate an important challenge affecting access for a primary health care system that should be free to users. Informal payments and corruption are reported at all levels [17]. Governance and stewardship is arguably the biggest challenge for BPHS services in Afghanistan. Conflict and insecurity also impedes implementation and monitoring. Disappointingly, in spite of having a well-defined and accepted package of primary health care services, these factors have challenged primary health care services in reaching the many vulnerable and marginalized families.

Overall, the GoIRA also needs to increase its funding to the health sector. Financing models from LMICs must also be explored, especially what Lagomarsino and colleagues call “national health insurance models that do not conform to historical archetypes” [18]. Many LMICs have large populations of people who are poor and have few resources to contribute. They also have informal economies making income tax deductions a challenge to implement. Diverse LMICs including Kenya, Rwanda, and Thailand have been able to increase public expenditure in health through a “mix of prepayment mechanisms” including allocations from general budget revenue, earmarked taxes, payroll deductions, and (to a lesser extent) household premium contributions. These funds have been used to improve governance and stewardship at the point of service, and limit formal and informal out-of-pocket expenditure and private household payments directly to health care providers [18]. Given the low level of public sector financing in Afghanistan and high out-of-pocket expenditure [9] health financing is a critical area for Afghanistan to assess.

Some LMICs stratify or prioritise ‘free of cost’ services using incremental pooled funding. It can be seen as a ‘safety net’ approach. It starts with different pools for different target populations and expands or combines them over time [18]. Some countries provide a safety net for poorer quintiles and mothers and children under five years. Many countries limit the number of risk pools and some countries have moved towards covering a greater target population, maintaining one pool if feasible. However, risk pools require significant administrative oversight which require their own governance and financing. Currently in Afghanistan this is not likely to work well. As described above, Afghanistan donor funds are pooled and BPHS services are purchased from NGOs (except in three trial provinces) and services are supposed to be free of cost for all people. However, as primary health care expenditure increases globally risk pools may soon be an imperative. As the Government of Afghanistan improves its stewardship functions and efficiency it may become a viable option.

A recent study on assessing the feasibility of introducing health insurance in Afghanistan concluded that while all stakeholders (including government, private sector, and development partners) were interested in introducing some form of risk pooling or health insurance, there were many challenges for the country to do so in the short term, including lack of clear legal guidance, poor governance, low quality of health services, and increasing insecurity [19].

## CONCLUSIONS

The WHO UHC definition intentionally focuses attention on a small number of critical areas. Experts in the area emphasize that principles of UHC are derived from rights and protection based literature, specifically: minimum core obligations; progressive realization; cost-effectiveness; shared responsibility; participatory decision-making; and prioritizing marginalized or vulnerable groups [2]. While more challenging to measure, these principles must also be considered in health programming in all low and middle income countries including Afghanistan.

Overall, health financing reform is a complex, long-term process that requires strong technical and management capacity, and commitment among stakeholders [20]. Given that Afghanistan has already applied the basic package of interventions model, it should continue progressing towards increasing public financing for health, decreasing OOP, and further development of its purchasing and stewardship functions.

Afghanistan’s investments in primary health care can be built upon and improved by strategies that will bring it closer to UHC [11]. With this aim, Afghanistan should also focus on areas suggested by Moreno-Serra & Smith [21] which can influence the effects of UHC (or progression towards it) on health outcomes. This includes a focus on quality of governance, stronger public sector administration and provider accountability, committing to UHC, improving the quality of services and ensuring a fairer distribution of access to care and health outcomes [21].

Afghanistan is among the most challenging places to build a well-functioning health system and introduce UHC. At the same time, it has technical and financial resources from many development partners, as well as reforms to improve governance throughout the country. While it is a challenging time for the country, it can also be a window of opportunity to plan for and start implementing and monitoring new ways to extend affordable care to more people particularly in conflict affected and other hard-to-reach areas.
